# A Pivotal Role for Tryptophan 447 in Enzymatic Coupling of Human Endothelial Nitric Oxide Synthase (eNOS): EFFECTS ON TETRAHYDROBIOPTERIN-DEPENDENT CATALYSIS AND eNOS DIMERIZATION*

**DOI:** 10.1074/jbc.M113.493023

**Published:** 2013-08-21

**Authors:** Matthew A. Benson, Helen Batchelor, Surawee Chuaiphichai, Jade Bailey, Hanneng Zhu, Dennis J. Stuehr, Shoumo Bhattacharya, Keith M. Channon, Mark J. Crabtree

**Affiliations:** From the § British Heart Foundation Centre of Research Excellence, Division of Cardiovascular Medicine, University of Oxford, John Radcliffe Hospital, Oxford OX3 9DU, United Kingdom,; the ‡ Nuffield Department of Clinical Medicine, Target Discovery Institute, The Wellcome Trust Centre for Human Genetics, Roosevelt Drive, Oxford OX3 7BN, United Kingdom, and; the ¶ Department of Pathobiology, Lerner Research Institute, Cleveland Clinic Foundation, Cleveland, Ohio 44195

**Keywords:** Endothelial Dysfunction, Nitric Oxide Synthase, Superoxide Ion, Tetrahydrobiopterin, Tryptophan, Superoxide, Uncoupling

## Abstract

Tetrahydrobiopterin (BH4) is a required cofactor for the synthesis of NO by NOS. Bioavailability of BH4 is a critical factor in regulating the balance between NO and superoxide production by endothelial NOS (eNOS coupling). Crystal structures of the mouse inducible NOS oxygenase domain reveal a homologous BH4-binding site located in the dimer interface and a conserved tryptophan residue that engages in hydrogen bonding or aromatic stacking interactions with the BH4 ring. The role of this residue in eNOS coupling remains unexplored. We overexpressed human eNOS W447A and W447F mutants in novel cell lines with tetracycline-regulated expression of human GTP cyclohydrolase I, the rate-limiting enzyme in BH4 synthesis, to determine the importance of BH4 and Trp-447 in eNOS uncoupling. NO production was abolished in eNOS-W447A cells and diminished in cells expressing W447F, despite high BH4 levels. eNOS-derived superoxide production was significantly elevated in W447A and W447F *versus* wild-type eNOS, and this was sufficient to oxidize BH4 to 7,8-dihydrobiopterin. In uncoupled, BH4-deficient cells, the deleterious effects of W447A mutation were greatly exacerbated, resulting in further attenuation of NO and greatly increased superoxide production. eNOS dimerization was attenuated in W447A eNOS cells and further reduced in BH4-deficient cells, as demonstrated using a novel split *Renilla* luciferase biosensor. Reduction of cellular BH4 levels resulted in a switch from an eNOS dimer to an eNOS monomer. These data reveal a key role for Trp-447 in determining NO *versus* superoxide production by eNOS, by effects on BH4-dependent catalysis, and by modulating eNOS dimer formation.

## Teaser

**Background:** Interaction of tetrahydrobiopterin (BH4) with a key tryptophan residue in the NOS active site is critical for activity.

**Results:** Mutation of tryptophan 447 causes eNOS uncoupling and monomerization.

**Conclusion:** Tryptophan 447 determines enzymatic coupling of human eNOS.

**Significance:** The development of BH4-based strategies to restore NOS function must consider the structural effects of BH4 binding and their role in NOS coupling.

## Introduction

NOSs catalyze the five-electron oxidation of l-arginine to produce l-citrulline and NO, an important signaling molecule in immunological, neuronal, and cardiovascular homeostasis and disease (1). In mammals, NO is generated by three distinct NOS isoforms, referred to as inducible (iNOS),^2^ neuronal, and endothelial (eNOS) (2). All NOS isoforms are obligate homodimers with similar structures. The N-terminal catalytic oxygenase domain (NOS_ox_) binds heme, the substrate l-arginine, and the essential pterin cofactor tetrahydrobiopterin (BH4), whereas the C-terminal reductase domain (NOS_red_) supplies electrons through bound flavin mononucleotide, flavin adenine dinucleotide, and NADPH. A central linker binds calmodulin and modulates the transfer of electrons from NOS_red_ of one subunit to the NOS_ox_ of the other dimer subunit (3–6).

BH4 is required for NO synthesis by all NOS isoforms. Although fully reduced tetrahydropterins support catalysis by NOSs, oxidized pterin species such as 7,8-dihydrobiopterin (BH2) and biopterin are catalytically incompetent, having the same allosteric effects without the ability to catalyze NO production (7). EPR studies showed that in the absence of BH4 (or presence of excess BH2), superoxide is the sole *in vitro* product of recombinant eNOS. In the absence of BH4, electron transfer from NOS flavins becomes “uncoupled” from l-arginine oxidation, the ferrous-dioxygen complex dissociates, and superoxide is released from the oxygenase domain (8, 9). This eNOS-derived superoxide production has been implicated in a wide variety of molecular, animal, and clinical models of vascular disease, including diabetes (10, 11), cigarette smoking (12), hypertension (13), and atherosclerosis (14).

**FIGURE 1.**
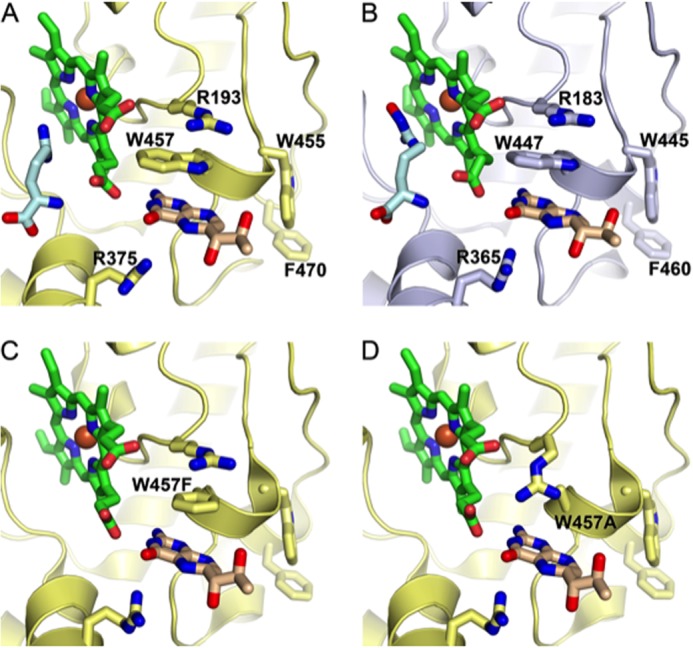
**Human eNOS and murine iNOS have closely related structures.** *A*, the active site of iNOS (*yellow*), showing the heme group (*green*) and arginine substrate (*pale blue*). *B*, the active site of eNOS (*gray*), demonstrating its close structural similarity with iNOS. Structural superposition of human eNOS and murine iNOS (PDB codes 3NOS and 1NOD, respectively) was performed using PDBe Fold. The top-ranked root mean square deviation between 365 matched residues in a single protein chain was 0.81Å. *C*, the active site of iNOS showing the W457F mutation. *D,* the active site of iNOS showing the W457A mutation. Figures were produced using PyMOL on the basis of Fig. 1 by Wang *et al.* (15) using PDB codes 3NOS (eNOS), 1NOD (iNOS), 1JWJ (iNOS W457F), and 1JWK (iNOS W457A).

As well as being pivotal in the transfer of electrons to the Fe(II)O_2_ complex and radical formation, the binding of BH4 to iNOS also has positive effects on dimerization and increases the binding affinity of the enzymatic substrate arginine. Previous studies looking at the binding of BH4 to iNOS have demonstrated that BH4 makes hydrogen bonds with the heme propionate and exhibits extensive interactions with the residues Trp-455, Trp-457, Phe-470, Arg-375, and Arg-193, as shown in Fig. 1. In particular, mutation of Trp-457 to Phe and Ala residues in iNOS greatly disrupts the interaction of Trp-457 with BH4 and decreases NO synthesis activity by 3.3-fold and 8-fold, respectively (15, 16). However, the role of BH4 binding in eNOS, the specific interaction of BH4 with this residue, and the role of eNOS Trp-447 (Trp-457 in iNOS) in enzymatic uncoupling remain unexplored.

eNOS uncoupling is often thought to occur in parallel with eNOS monomerization, and confusion exists as to whether changes in the dimer/monomer ratio are directly related to the functional uncoupling of eNOS because current literature suggests that only the dimeric form of eNOS is biochemically active and able to generate either NO or superoxide (17). Questions also remain as to whether eNOS in the uncoupled state exists as a monomer, therefore suggesting that the influences of BH4 on “dimer stabilization” and the coupling of eNOS are not necessarily one and the same effect.

Accordingly, we sought to elucidate a mechanistic role for the interaction of BH4 with eNOS Trp-447 in the regulation of eNOS uncoupling and monomerization. To address these questions, we expressed eNOS W447F and eNOS W447A mutants in HEK293 cells and in cells that stably express doxycycline-regulatable GTPCH protein to determine the effects of high or low intracellular BH4. We also developed a novel biosensor of eNOS dimerization on the basis of the reconstitution of split *Renilla* luciferase, revealing for the first time that the Trp-447 residue within the BH4 binding site of eNOS is required for efficient NO production by the enzyme, is critical for the coupling of eNOS, and also plays a role in dimerization. These findings have significant consequences for the therapeutic potential of BH4-dependent eNOS catalysis and dimerization.

## EXPERIMENTAL PROCEDURES

### 

#### 

##### Molecular Modeling of the eNOS Active Site

Figures were produced using PyMOL, on the basis of Fig. 1 by Wang *et al.* (15) using PDB codes 3NOS (eNOS), 1NOD (iNOS), 1JWJ (iNOS W457F), and 1JWK (iNOS W457A).

##### Generation of Tet-regulatable Cells

We used National Institutes of Health 3T3 murine fibroblasts stably transfected with a Tet-Off transactivator construct as described previously (18). In the presence of doxycycline, binding of the transactivator is blocked, and gene expression is prevented. These 3T3-Tet-Off cells, previously shown not to express GTPCH (19) and also confirmed to be devoid of eNOS protein, were stably transfected with a plasmid encoding hemagglutinin antigen-tagged human GTPCH under the control of a tetracycline-responsive element. Individual colonies were isolated and analyzed for GTPCH expression, and a cell line termed “GCH cells” was established from expansion of a single colony. GCH/eNOS cells were produced by stable transfection of GCH cells with a plasmid encoding a human eNOS-eGFP fusion protein as described (20). In this model, addition of doxycycline to GCH/eNOS cells results in diminished GCH mRNA, GTPCH protein, and BH4 levels, leading to uncoupling of eNOS (18).

##### Measurement of eNOS Protein Levels by eGFP Fluorescence

Cell pellets were lysed in phosphate-buffered saline containing 1 mmol/liter dithioerythritol and 100 μmol/liter EDTA as for BH4 analysis. Sample fluorescence was quantified using a TEKAN fluorescence plate reader and a standard curve generated using recombinant eGFP. Because recombinant eNOS was expressed as an eNOS-eGFP fusion protein, eGFP fluorescence and eNOS levels were directly proportional.

##### Generation of Tryptophan eNOS Mutants

QuikChange site-directed mutagenesis (Stratagene) was used to create W447A, W447F, and C99A human eNOS mutants. Mutant primers were designed and used as follows: W447A, 5′-TGCAGACTGGGCCGCGATCGTGCCCCCC-3′ (sense) and 5′-GGGGGGCACGATCGCGGCCCAGTCTGCA-3′ (antisense); W447F, 5′-GCAGACTGGGCCTTCATCGTGCCCCCCA-3′ (sense) and 5′-TGGGGGGCACGATGAAGGCCCAGTCTGC-3′ (antisense); and C99A, 5′-CACCCCAAGACGCGCCCTGGGCTCCCTG-3′ (sense) and 5′-CAGGGAGCCCAGGGCGCGTCTTGGGGTG-3′ (antisense). The sequence of each eNOS single and double mutant was confirmed by DNA sequencing (SourceBioscience, UK). Mutant constructs were transiently expressed in GCH cells using FuGENE 6 (Roche).

##### Cell Culture

Cells were cultured in DMEM (Invitrogen) supplemented with 2 mm glutamine, 100 units/ml penicillin, and 0.1 mg/ml streptomycin. Additionally, GCH cells were maintained using media containing the antibiotics hygromycin (200 μg/ml) and genetecin (200 μg/ml), whereas GCH/eNOS-eGFP cell medium also included 2 μg/ml puromycin. Where appropriate, 1 μg/ml doxycycline was added to cell culture medium to abolish transcription of GCH1 mRNA.

##### Monomer and Dimer Western Blotting

Low-temperature SDS-PAGE was performed for detection of the eNOS monomer and dimer. Briefly, cells lysates were prepared by homogenization in ice-cold CelLytic M buffer (Sigma) containing protease inhibitor (Roche Applied Science) and subjected to three freeze-thaw cycles in liquid nitrogen. Lysates were centrifuged at 13,200 rpm for 10 min at 4 °C, and samples were prepared using Laemmli sample buffer (Sigma). Protein lysates were resolved using a 6% Tris-glycine gel (Invitrogen) under reducing conditions. All gels and buffers were pre-equilibrated to 4 °C before electrophoresis, and the buffer tank was placed in an ice bath during electrophoresis to maintain the gel temperature below 15 °C. Standard blotting techniques were used, and membranes were incubated with mouse anti-eNOS polyclonal antibody (BD Transduction Laboratories) as described previously (18, 21).

##### Creation of eNOS Dimerization Biosensors

Rluc8.1 and 8.2 fragments were PCR-amplified using the rluc8 plasmid as a template (a gift from Prof. Sanjiv Gambhir, Stanford University). The rluc8 PCR fragments were cloned into pcDNA3. Gateway destination plasmids were then created by cloning the vector conversion kit (Invitrogen) into the rluc8.1 and rluc8.2 vectors. For the creation of stable cell lines, this destination cassette was cloned into the pIRES-puro and Neo plasmids (Clontech). pENTR clones encoding our genes of interest were either created by PCR or purchased from Geneservice or Thermo Fisher. To create expression plasmids, pENTR clones were recombined with the destination vectors using LR clonase according to the instructions of the manufacturer. Recombinant Rluc8 expression plasmids and a LacZ-encoding control plasmid were then transfected into HEK293T or GCH cells and grown on white 96-well plates using FuGENE HD (Roche). We placed our rluc8.1 and rluc8.2 halves onto either end of eNOS. rluc8.1 was added to the C terminus (reductase domain), and rluc8.2 was added to the N terminus (oxygenase domain).

##### Detection of Biosensor Luminescence by Protein Fragment Complementation Assay

24 h following transfection, cells were treated with drugs as indicated. Cells were then washed with PBS, and reconstituted Rluc8 activity was measured using benzyl-coelenterazine (Nanolight) on a BMG Polarstar plate reader (5-s read time). Cells were lysed by adding LacZ lysis buffer (120 μm TrisPO4 (pH 7.8), 10 mm 1,2-cyclohexylenedinitrilotetraacetic acid, 30% glycerol, and 1% Triton X-100) to each well, and LacZ activity was measured using ortho-nitrophenyl-β-galactopyranoside. Rluc8 readings were normalized using LacZ activity.

##### Analysis of NO Synthesis by eNOS

Cellular NO synthesis by eNOS was assessed by measuring the conversion of ^14^C l-arginine to citrulline with HPLC detection, in the presence and absence of NG-monomethyl-l-arginine, as described previously (22).

##### Quantification of Superoxide Production by HPLC

Measurement of 2-hydroxyethidium formation by HPLC was used to quantify superoxide production, as described previously (23, 24). Cells were washed three times in PBS and incubated in Krebs-Hepes buffer in the presence or absence of 100 μm L-NAME. After 30 min, 25 μm dihydroethidium was added, and cells were incubated for an additional 20 min at 37 °C. Cells were then harvested by scraping, centrifuged, and lysed in ice-cold methanol. 100 mm hydrochloric acid was added (1:1 v/v) prior to loading into the autosampler for analysis. All samples were stored in darkened tubes and protected from light at all times. Separation of ethidium, oxyethidium, and dihydroethidium was performed using a gradient HPLC system (Jasco) with an ODS3 reverse phase column (250 mm, 4.5 mm; Hichrom) and quantified using a fluorescence detector set at 510 nm (excitation) and 595 nm (emission). A linear gradient was applied from mobile phase A (0.1% TFA) to mobile phase B (0.1% TFA in acetonitrile) over 23 min (30–50% acetonitrile).

##### Biopterin Quantification by HPLC with Electrochemical Detection

BH4, BH2, and biopterin levels in cell lysates were determined by HPLC, followed by electrochemical and fluorescent detection, as described previously (18, 25). Briefly, cells were grown to confluency and harvested by trypsinization. Sample pellets were resuspended in 50 mm phosphate-buffered saline (pH 7.4) containing 1 mm dithioerythritol and 100 μm EDTA and subjected to three freeze-thaw cycles. Following centrifugation (15 min at 13,000 rpm and 4 °C), samples were transferred to new, cooled microtubes and precipitated with 1 m cold phosphoric acid, 2 m TCA, and 1 mm dithioerythritol. Samples were mixed vigorously and then centrifuged for 15 min at 13,000 rpm and 4 °C. Samples were injected onto an isocratic HPLC system and quantified using sequential electrochemical (Coulochem III, ESA Inc., UK) and fluorescence (Jasco) detection. HPLC separation was performed using a 250-mm ACE C-18 column (Hichrom) and a mobile phase comprising 50 mm sodium acetate, 5 mm citric acid, 48 μm EDTA, and 160 μm dithioerythritol (pH = 5.2) (all ultrapure electrochemical HPLC grade) at a flow rate of 1.3 ml/min. Background currents of +500 μA and −50 μA were used for the detection of BH4 on electrochemical cells E1 and E2, respectively. 7,8-BH2 and biopterin were measured using a Jasco FP2020 fluorescence detector. Quantification of BH4, BH2, and biopterin was done by comparison with authentic external standards and normalized to sample protein content.

##### Statistical Analysis

Data are presented as mean ± S.E. Data were subjected to the Kolmogorov-Smirnov test to determine distribution. Groups were compared using Mann-Whitney *U* test for non-parametric data or Student's *t* test for parametric data. When comparing multiple groups, data were analyzed by analysis of variance with Newman-Keuls post-test for parametric data or Kruskal-Wallis test with Dunn's post-test for non-parametric data. A value of *p* < 0.05 was considered statistically significant.

## RESULTS

### 

#### 

##### Characterization of Mutant eNOS Expression

**FIGURE 2.**
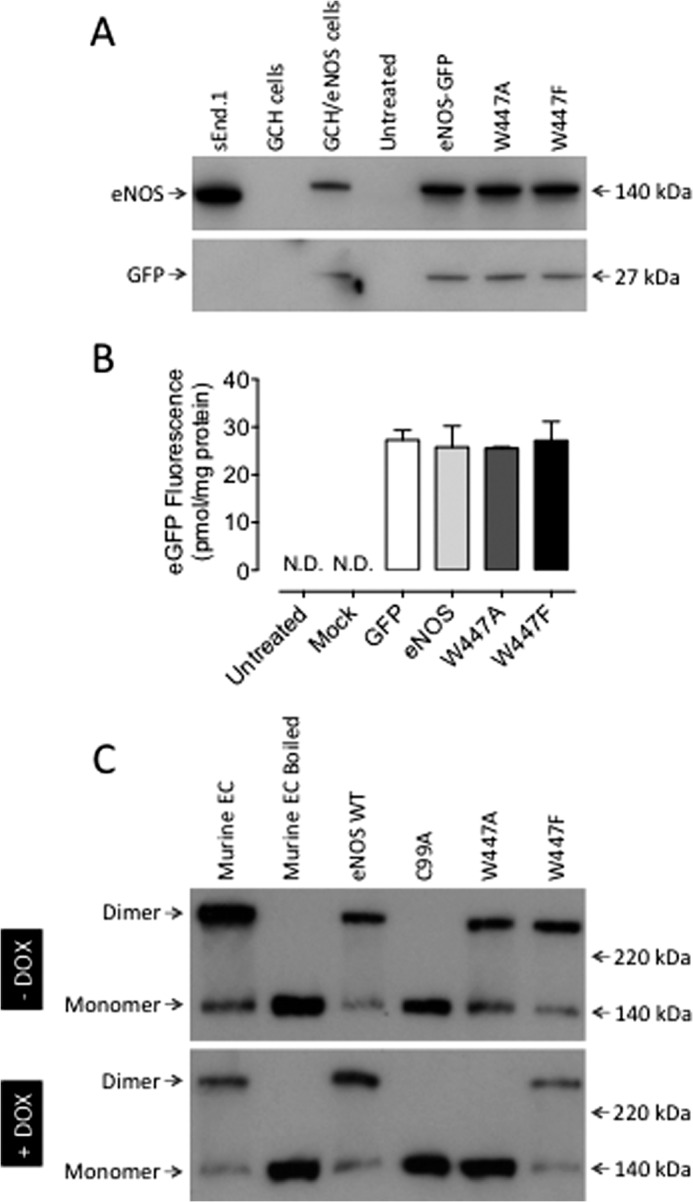
**Overexpression of eNOS Trp-447 mutants results in monomerization and altered localization of eNOS protein.** GCH cells were cultured in the presence or absence of DOX for 7 days and then transiently transfected with wild-type eNOS, W447A or W447F mutant eNOS DNA. *A*, Western blot analysis showed equal expression of our wild-type and mutant eNOS constructs using both anti-eNOS and anti-GFP antibodies. sEnd.1 murine endothelial cells and cells stably expressing eNOS-GFP were used as controls. *B*, equivalent expression levels were demonstrated by fluorescence quantification. *C*, eNOS dimerization was compared by low temperature Western blotting in cells in the absence (high BH4) or presence (low BH4) of DOX. Only the C99A mutation of eNOS resulted in significant monomerization compared with endothelial cell (*EC*) cold and boiled sample controls. In contrast, in BH4-deficient cells, following DOX treatment, the eNOS dimer was totally abolished in W447A and significantly attenuated in W447F mutant-expressing cells. These changes were linked with increases in detectable levels of eNOS monomer. Western blot analyses are representative of three separate experiments. *N.D.* = not detectable.

We first investigated the effect of Trp-447 mutation on eNOS expression in murine fibroblasts. Western blotting confirmed that transient expression of wild-type and mutant eNOS was equal (Fig. 2*A*), as quantified by GFP fluorescence (*B*). The effect of Trp-447 mutation on eNOS dimerization was assessed by immunoblotting, and the ratio of eNOS monomer/dimer was quantified in GCH cells in the presence and absence of doxycycline, generating low or high levels of BH4. As shown in Fig. 2*C*, in conditions of high BH4 levels, we observed that human eNOS and the respective Trp-447 mutants existed almost entirely as dimers, as did a murine eNOS-positive control. As a positive control for monomerization, expression of C99A resulted in complete loss of the eNOS dimer, perhaps because of disruption of Zn coordination, as described previously (26). In doxycycline-treated, BH4-deficient cells, W447A eNOS was predominantly monomeric, and the amount of dimer in the W447F mutant was also decreased significantly (Fig. 2*C*).

##### Mutation of Trp-447 Uncouples eNOS

**FIGURE 3.**
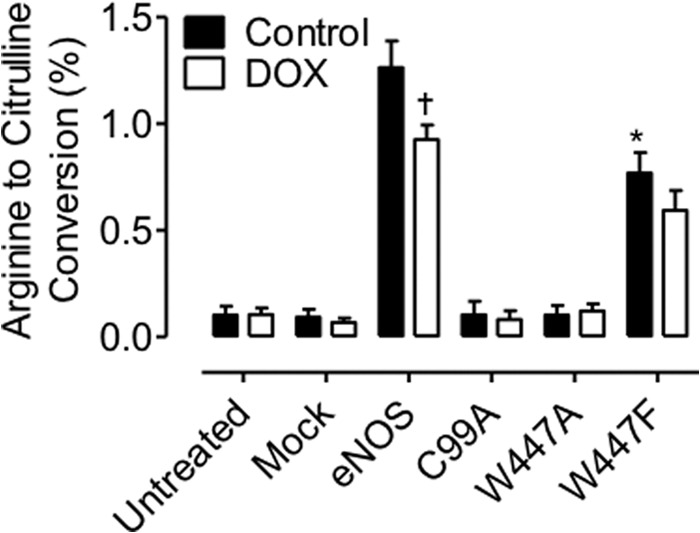
**Trp-447 mutation decreases eNOS activity.** GCH cells were cultured in the presence or absence of DOX for 7 days and then transiently transfected with wild-type eNOS, W447A, or W447F mutant eNOS DNA. eNOS activity, as assessed by measuring the conversion of radiolabeled arginine to citrulline, was significantly attenuated in BH4-deficient, DOX-treated cells (†, *p* < 0.05). Expression of C99A or W447A in cells containing either high, or low levels of BH4 totally abolished eNOS activity (*, *p* < 0.001) with no further effect of doxycycline. W447F mutation resulted in an attenuation of eNOS activity in cells containing high levels of BH4 with a further significant decrease observed in BH4-deficient, DOX-treated cells (†, *p* < 0.05). *n* = 6.

We next sought to establish the effect of Trp-447 mutation on eNOS uncoupling and subsequent NO and superoxide production. eNOS activity, as assessed by measuring the conversion of radiolabeled arginine to citrulline, was significantly attenuated in BH4-deficient, DOX-treated cells (Fig. 3; †, *p* < 0.05), as described previously (18). Expression of C99A or W447A in cells containing either high or low levels of BH4 totally abolished eNOS activity (*p* < 0.001) with no further effect of doxycycline. In contrast, W447F mutation resulted in an attenuation of eNOS activity in cells containing high levels of BH4, with a further significant decrease observed in BH4-deficient, DOX-treated cells (*p* < 0.05).

**FIGURE 4.**
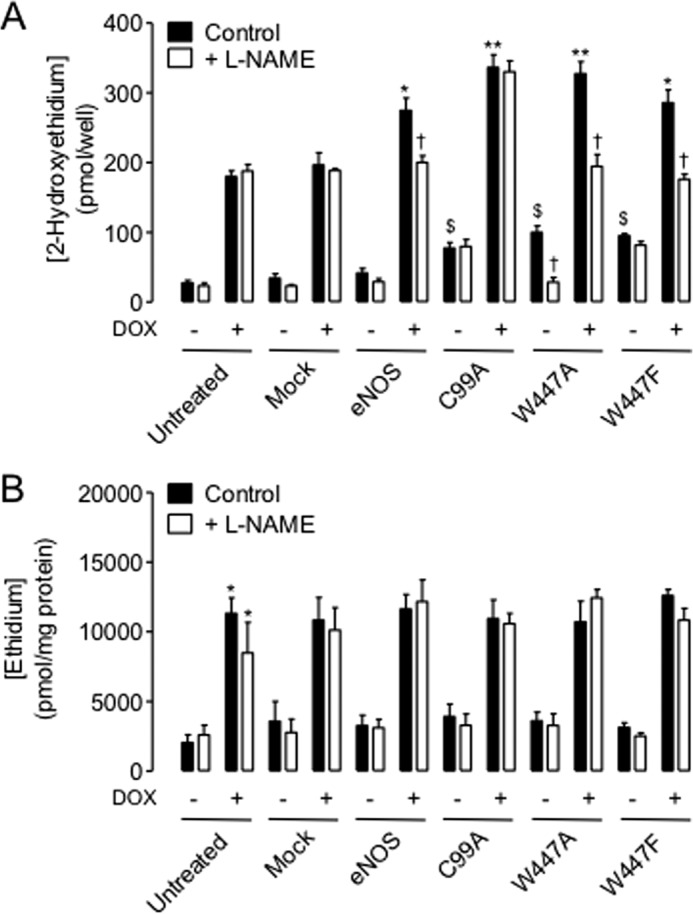
**eNOS-derived superoxide production is exacerbated by Trp-447 mutation in a BH4-dependent manner.** GCH cells were cultured in the presence or absence of DOX for 7 days and then transiently transfected with wild-type eNOS, W447A, or W447F mutant eNOS DNA. Accumulation of 2-hydroxyethidium following exposure of cells to dihydroethidium was used as an indirect measure of superoxide production and was quantified by HPLC. *A*, in high BH4 conditions (no DOX), eNOS overexpression had no effect on superoxide production. C99A, W447A, and W447F mutation increased the detectable levels of 2-hydroxyethidium ∼2.5-fold ($, *p* < 0.05), which was only inhibitable with L-NAME in W447A but not C99A or W447F mutant cells (†, *p* < 0.05). DOX exposure elevated superoxide levels in untreated and mock control cells, as described previously (18). However, in these low-BH4 cells, levels of 2-hydroxyethidium were elevated in wild-type and mutant cells (wild type < Trp-eNOS-Phe < W447A) (*, *p* < 0.05; **, *p* < 0.01). This elevation in 2-hydroxyethidium accumulation was totally abolished following L-NAME treatment in Trp-447 but not C99A mutant cells. *n* = 6. *B*, ethidium accumulation was used as an indicator of overall oxidative stress. DOX treatment strikingly elevated the levels of ethidium within cells with no additional effect of wild-type or mutant eNOS transfection. *, *p* < 0.05, *n* = 6.

To further investigate the enzymatic uncoupling of eNOS induced by mutation at Trp-447, we next investigated the ability of eNOS to produce superoxide using dihydroethidium fluorescence. eNOS-derived superoxide was distinguished using the arginine analog L-NAME. As described previously, prevention of BH4 production upon doxycycline exposure of untreated cells caused a striking elevation of superoxide production in an eNOS-independent manner (*p* < 0.001). We hypothesize that this demonstrates the general antioxidant role of BH4 within the cell. Furthermore, although having no effect in BH4-replete cells, eNOS overexpression in BH4-deficient cells increased the production of superoxide in an L-NAME-inhibitable manner, indicating eNOS uncoupling (*p* < 0.05). In contrast, W447A mutation significantly elevated eNOS-derived superoxide production in cells with high BH4 levels and in BH4-deficient cells (*p* < 0.05). The levels of superoxide produced by the W447A mutant eNOS were significantly greater than those produced by wild-type eNOS (*p* < 0.05). The increased production of superoxide observed in W447F-expressing cells was not inhibitable with L-NAME in BH4-replete cells but totally abolished by L-NAME in cells containing low levels of BH4. In contrast to the differences in 2-hydroxyethidium, specific for superoxide production, the accumulation of ethidium (an indicator of production of other reactive oxygen species) remained unchanged in eNOS wild-type or mutant-expressing cells (Fig. 4).

**FIGURE 5.**
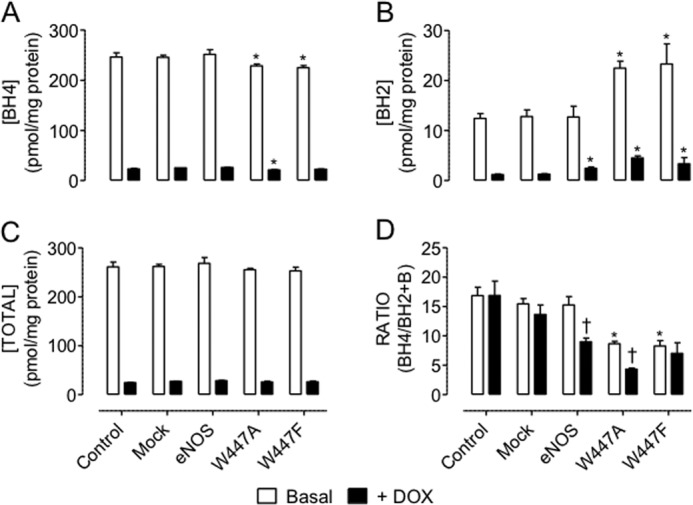
**Mutant eNOS-derived superoxide exacerbates BH4 oxidation.** GCH cells were cultured in the presence or absence of DOX for 7 days and then transiently transfected with wild-type eNOS, W447A, or W447F mutant eNOS DNA. Intracellular biopterins were measured using HPLC as described under “Experimental Procedures.” BH4 (*A*) was oxidized to BH2 (*B*) by mutant but not wild-type eNOS-derived superoxide in high BH4-containing cells. Following induction of BH4 deficiency with DOX, eNOS wild-type expression also triggers BH4 oxidation to BH2. *C*, total biopterin levels remains unaffected by eNOS overexpression. The BH4:BH2 ratio is attenuated by uncoupled eNOS (†, *p* < 0.05) because of DOX treatment and greatly exacerbated in W447A *versus* W447F eNOS overexpression (**, *p* < 0.01), suggesting that W447A mutation worsens enzymatic uncoupling compared with wild-type eNOS (*n* = 4).

BH4 was only oxidized to BH2 by superoxide produced from wild-type eNOS when the total levels of BH4 were low and insufficient to efficiently couple the enzyme. eNOS wild-type overexpression had no effect on BH4 homeostasis when total levels of BH4 were saturating. Intracellular BH4 was markedly oxidized to BH2 by superoxide produced from W447A and W447F eNOS mutants under conditions of both high and low levels of BH4. This oxidation of BH4 in W447A eNOS-expressing cells with high BH4 levels was comparable with that observed in WT eNOS in low BH4 conditions. In BH4-deficient cells, oxidation of BH4 and the accumulation of BH2 were further exacerbated in W447A eNOS but not either W447F eNOS or wild-type eNOS, as revealed by the diminished ratio of BH4/BH2 in cells expressing W447A eNOS following doxycycline exposure (Fig. 5).

Overall, the diminished NO production, elevated superoxide production, and the accumulation of BH2 suggest that the W447A and W447F mutants uncouple eNOS independently of total BH4 levels. However, the effects of W447A mutation are exacerbated when levels of BH4 are very low, resulting in a worsening of eNOS uncoupling.

##### The eNOS Dimerization Biosensor Reveals That Trp-447 Mutation Causes Monomerization in BH4 Deficiency

**FIGURE 6.**
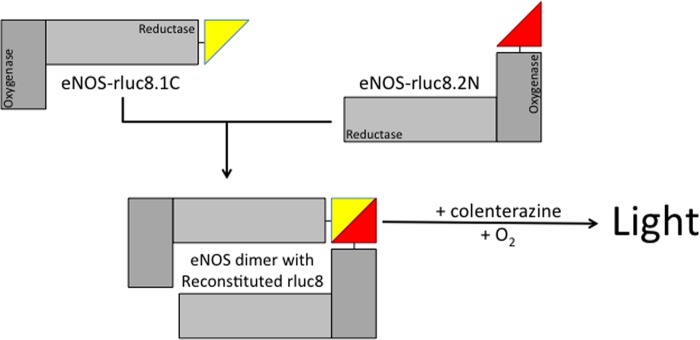
**Novel *Renilla* luciferase biosensor of eNOS dimerization.** We developed a novel protein-protein interaction assay on the basis of the reconstitution of split *Renilla* luciferase (rluc8). eNOS-separate constructs were generated to express eNOS tagged with Rluc-8.1on the N terminus and Rluc-8.2 on the C terminus. Cotransfection of these two constructs allows dimerization. Following the addition of coelenterazine substrate, the magnitude of dimerization was quantified by protein fragment complementation assay and luminescence as described under “Experimental Procedures.”

**FIGURE 7.**
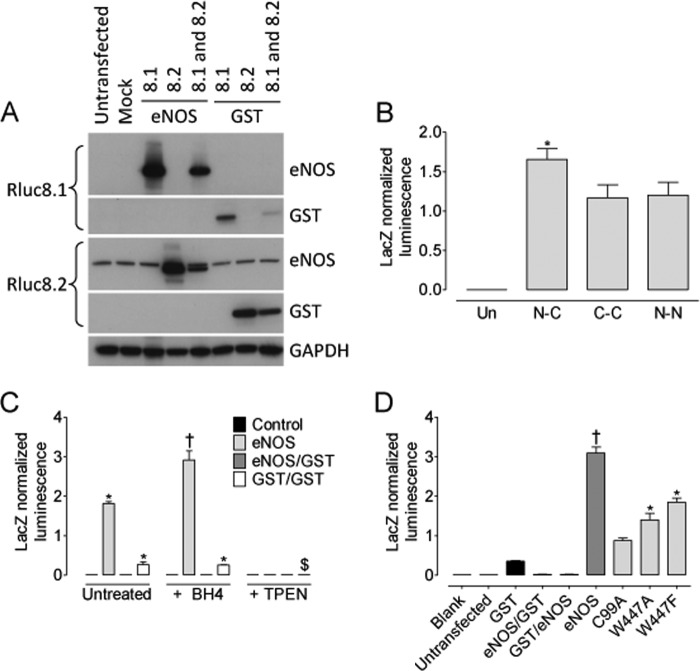
**Overexpression of W447A and W447F results in dramatic monomerization of eNOS.** HEK cells were transfected with both eNOS-Rluc constructs as well as GST-Rluc control plasmids. *A*, characterization of plasmid overexpression was done by Western blotting using specific eNOS and Rluc antibodies. *B*, different rluc biosensor constructs were tested where rluc8.1 and rluc 8.2 were placed on the N- and C terminus (*N-C*), the C- and C terminus (*C-C*), or the N- and N terminus (*N-N*) on each eNOS construct, respectively. *Un* = untransfected; *, *p* < 0.05 *versus* C-C and N-N. *C*, transfected cells were then subjected to treatment with either BH4 or *N,N,N,N*-tetrakis(2-pyridylmethyl)ethylenediamine (*TPEN*) and eNOS dimerization compared with control GST-overexpressing cells. Overexpression of both eNOS-Rluc8.1 and eNOS-Rluc8.2 revealed a robust luminescence signal. eNOS dimerization only, not GST dimerization, was significantly increased by BH4 treatment (†, *p* < 0.05, *, *p* < 0.01 *versus* control, $, *p* < 0.05 *versus* GST expressing cells). Sequestration of intracellular zinc with TPEN abolished all dimerization in eNOS- and GST-expressing cells (*n* = 3). *D*, eNOS dimerization is prevented in C99A mutants and significantly attenuated in HEK cells expressing W447A and W447F mutant eNOS. *, *p* < 0.01; *n* = 3.

**FIGURE 8.**
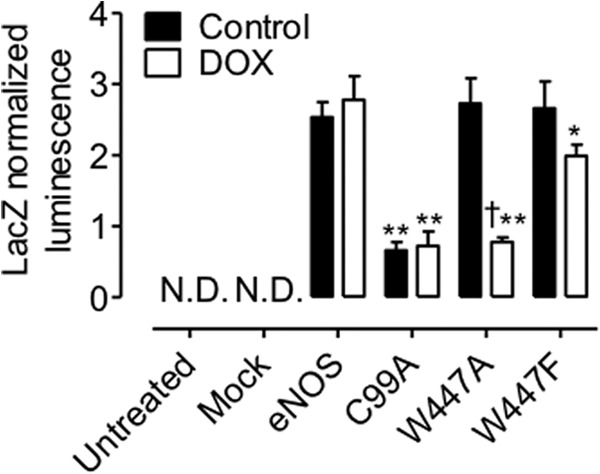
**BH4 deficiency exacerbates monomerization of eNOS.** GCH cells were cultured in the presence or absence of DOX for 7 days and then transiently transfected with either wild-type, W447A, or W447F mutant eNOS in the Rluc constructs, as described previously. Dimerization was again assessed by protein fragment complementation luminescence assay. Modulation of BH4 levels by DOX had no effect on eNOS dimerization. In contrast, W447A and W447F dramatically inhibited the ability of eNOS to dimerize (*, *p* < 0.05; **, *p* < 0.001). W447A eNOS dimerization was significantly lower than W447F (†, *p* < 0.05). C99A mutation resulted in significantly attenuated dimerization of eNOS (**, *p* < 0.001; *N.D.*, none detectable with a limit of detection of 2 × 10^5^ RLU, equal to 0.02 on the *y* axis. *n* = 4.

Having established that Trp-447 is critical for efficient NO production from eNOS and that its mutation initiated superoxide production rather than NO, was important to determine whether these changes in signaling of eNOS were because of changes in dimerization. To this end, we developed a novel biosensor on the basis of the reconstitution of *Renilla* luciferase as depicted in Fig. 6. The eNOS dimerization biosensor was created from the rluc8 construct as described under “Experimental Procedures.” We first characterized the biosensor by Western blotting and luminometry. Following transient transfection with either the experimental eNOS-Rluc fusion constructs or a GST control biosensor, HEK293 cells were shown to express either eNOS-Rluc8.1, eNOS-Rluc8.2, GST-Rluc8.1, or GST-Rluc8.2, where appropriate, using Rluc8.1- or Rluc8.2-specific antibodies (Fig. 7*A*). We placed our rluc8.1 and rluc8.2 halves onto either end of eNOS. rluc8.1 was added to the C terminus (reductase domain), and rluc8.2 was added to the N terminus (oxygenase domain). This was done using two individual constructs, with eNOS tagged only at one end at a time. Cells were then transfected with both constructs, either tagged at the N or C terminus, and luminescence was measured by protein fragment complementation assay. During the setup and initial characterization of our system, we tested the ability of luciferase to generate a detectable signal when eNOS-rluc8.1N and eNOS-rluc8.2N or eNOS-rluc8.1C and eNOS-rluc8.2C were co-overexpressed. A detectable signal was generated from both “N-” or “C-overexpressed” constructs that was significantly smaller than that obtained from eNOS-rluc8.1C and eNOS-rluc8.2N overexpression (Fig. 7*B*). In cells expressing both the Rluc8.1 and Rluc8.2 fusion proteins, experiments were conducted to assess the effect of BH4-treatment on eNOS dimer formation. These data were then compared with that from the removal of intracellular zinc using *N,N,N,N*-tetrakis(2-pyridylmethyl)ethylenediamine (TPEN) as a positive control for monomerization. In HEK293 cells, control GST-Rluc and eNOS-Rluc, biosensor expression, and subsequent reconstitution were detected. As an important control to rule out nonspecific reconstitution of *Renilla* luciferase, no luminescence was detected when eNOS-Rluc8.1 and GST-Rluc8.2 were coexpressed (or *vice versa*, data not shown). Following exposure to BH4 (10 μm, 4 h), a significant increase in luminescence and, hence, dimer formation was observed in eNOS-Rluc but not control or GST-Rluc-expressing cells. Removal of intracellular Zinc, required for the dimerization of both eNOS and GST, abolished the luminescent signal (Fig. 7*C*). The next objective was to specifically investigate whether the Trp-447 residue within eNOS has any role in determining eNOS dimerization. The robust signal generated by eNOS-Rluc was dramatically attenuated following expression of C99A-mutant eNOS, described previously to be required for eNOS dimerization (26). The W447A and W447F eNOS mutants both exhibited a marked attenuation of the luminescence signal and, therefore, eNOS dimerization in the order C99A < W447A < W447F < WT-eNOS (Fig. 7*D*, *, *p* < 0.05). The ability of W447A and W447F mutation of eNOS to determine eNOS coupling and the effect of total intracellular BH4 on eNOS dimerization was next determined in GCH-Tet cells where intracellular BH4 deficiency was induced by doxycycline exposure. Mutation of eNOS-W447 in cells with saturating levels of BH4 had no effect on eNOS dimerization. In contrast, W447A and W447F overexpression in BH4-deficient cells markedly decreased eNOS dimerization in a graded manner, with W447A having a more significant effect than W447F (Fig. 8, **, *p* < 0.001 and *, *p* < 0.05, respectively).

## DISCUSSION

In this study, we investigated the role of Trp-447 in human eNOS uncoupling and reveal for the first time that Trp-447, situated within the BH4 binding site, is critical for the enzymatic coupling of eNOS and, therefore, efficient NO production by the enzyme. Moreover, mutation of this key residue results in an attenuated interaction of BH4 with eNOS and substantial superoxide production. We also use novel biosensors to demonstrate the effect of mutation of this residue on eNOS dimerization. This study identifies a pivotal role for Trp-447 and reveals new aspects of the relationship between eNOS uncoupling and dimerization, two independent mechanisms of eNOS activity and regulation.

The major findings of this study are as follows. First, mutation of W447A prevents eNOS dimer formation in low but not high BH4-containing cells. Second, W447A expression effectively abolishes, whereas W447F significantly attenuates, NO production from eNOS independently of BH4 levels. These effects are exacerbated when BH4 levels are diminished. Third, these changes in eNOS activity are associated with changes in superoxide production and BH4 oxidation. Finally, discordance exists between superoxide production and the monomerization of eNOS, thus suggesting that both the eNOS monomer and dimer are significant sources of superoxide following mutation of Trp-447. Interestingly, this source of superoxide production appears to switch from eNOS dimers in replete BH4 conditions to eNOS monomers when BH4 levels are deficient. Taken together, these findings provide clear evidence to support an important catalytic role for Trp-447 in the regulation of eNOS coupling and eNOS dimerization.

These findings provide important insights into the role of BH4 in regulating eNOS activity and eNOS coupling, which is well established to occur in a variety of *in vitro*, cell culture, and animal models of vascular disease (27). Previous studies have used models of BH4 deficiency to investigate vascular diseases such as hypercholesterolemia, hypertension, and diabetes (12, 28–33) and showed that many pathophysiological effects can be effectively rescued using BH4 supplementation either by pharmacological or genetic intervention. This is the first study to specifically investigate the impact of altering the interaction of BH4 with the eNOS active site on eNOS coupling rather than modulation of BH4 levels *per se*, therefore eliminating nonspecific effects of reduced BH4 levels on systems where BH4 also plays important regulatory roles in a NOS-independent manner, such as other intracellular antioxidant or redox effects.

We show a critical role for Trp-447 in eNOS function and further advance previous work on the basis of the equivalent Trp-457 mutation in mouse iNOS where possible enzymatic uncoupling was not addressed. Structural studies of the iNOS_oxy_ domain distinguish functional roles of BH4 that are modulated by Trp-457 (the Trp-447 equivalent in eNOS). We hypothesized that induction of eNOS uncoupling because of the mutation of W447A is due to altered BH4 binding to the enzyme. However, although W457A and W457F mutation in iNOS abolishes or inhibits NO synthesis, crystallographic structures demonstrate how W457A and W447F iNOS mutation does not result in dramatic changes in wild type position, orientation, and hydrogen bonding of BH4 (16). However, the structural basis for changes in dimer formation and iNOS function observed result from the rearrangement of Arg-193 following W457A mutation and the ability of Arg-193 to fill the open space left by replacement of the large tryptophan with the small arginine residue. In the W457A mutant iNOS_ox_ structure, the rearrangement of positively charged Arg-193 to form a T-shaped π-cation interaction with BH4 further stabilizes protein binding to BH4 while further destabilizing protein binding to the pterin radical that forms during catalysis. When BH4 forms the cation radical, as suggested by the eNOS_ox_ crystallographic structure and by EPR experiments, the protein binding of BH4 would be further destabilized by repulsive positive charge distribution between Arg-193 and the cofactor. Consistent with these predictions on the basis of the crystallographic structures, the rate of radical formation is further decreased or remains unchanged, whereas the rate of radical decay is further increased in W457A iNOS_ox_ relative to the W457F mutant, revealing that Trp-457 is implicated in the regulation of electron transfer during NO synthesis. Trp-457 mutants of iNOS in the presence of BH4 and l-Arg lead to reduced BH4 and l-Arg affinity and slower synthesis of NO (15). Full-length W457A iNOS, as well as the W457A and W457F iNOS_ox_ mutants, all exhibit diminished rates of NO synthesis and are uncoupled with respect to enzyme NADPH oxidation. NO synthesis activities of full-length and corresponding neuronal NOS mutants W678L and W678H (34) were less than the wild-type ones, and iNOS W457 mutations primarily slowed the rate of BH4 radical formation and sped up radical decay. Slower electron transfer from BH4 to Fe(II)O_2_ could uncouple NOS oxygen activation from NO synthesis if Fe(II)O_2_ decay is sufficiently fast in full- length NOS (35). In such a circumstance, superoxide release from the Fe(II)O_2_ intermediate would occur at the expense of NADPH oxidation and would uncouple NADPH oxidation from NO synthesis in NOS.

Because of the lack of eNOS mutant crystal structures, we assume the same electronic and structural effect of the Trp-447 mutant to be true in eNOS. For the first time, we present that Trp-447 plays an important role in dimer assembly in low BH4 conditions, more similar to neuronal NOS W678L mutation in the presence of both BH4 and l-Arg, where only 15% of the purified enzyme is dimeric and a drastically diminished production of NO is observed (34). As with neuronal NOS, mutation of Trp-447 did not result in totally abolished dimer formation, therefore suggesting that BH4 is not an absolute requirement for dimerization.

The BH4 dependence of dimer formation and superoxide production by W447A and W447F mutants differs compared with wild-type eNOS. The W447A mutation is more sensitive to intracellular BH4 concentration than either W447F or wild-type eNOS, respectively. Evidence for this comes from the different magnitude of superoxide production in altered BH4 conditions. In cells containing high levels of BH4, an elevation in eNOS mutant-derived superoxide production occurs with no concurrent change in eNOS dimer formation. In contrast, DOX-induced BH4 deficiency increases superoxide to a greater extent but with a corresponding decrease (WT > W447F > W447A) in dimerization, suggesting substantial monomer-derived superoxide. This supports previous reports that indicate that BH4 deficiency-induced eNOS uncoupling occurs simultaneously with monomerization. Studies of high glucose-treated endothelial cells (21, 37) and diabetic mice (38) reveal BH4 oxidation, superoxide production with a simultaneous increase in eNOS monomer. This is in discordance with other publications that state that the eNOS dimer is required for superoxide production. The inhibition of superoxide production from the eNOS monomer by L-NAME is somewhat confusing because all superoxide would be derived from the reductase domain and would, therefore, not be expected to be inhibited with L-NAME.

We hypothesize that there is a small amount of electron flow in the eNOS monomer that enables some degree of superoxide production from its reduced flavins. However, there is no precedent to expect that this monomer would be able to transfer electrons to its heme or would generate more superoxide per mole than a BH4-free eNOS dimer. As we report here, the superoxide produced by both W447F and W447A when BH4 levels are limiting occur along with a decrease in dimer formation, and the superoxide production became mostly inhibitable by L-NAME. We hypothesize that, in Trp-447 mutant cells in this circumstance, the bulk of the superoxide is produced from the remaining dimer (as evidenced by the background dimerization detected with our biosensor and by Western blotting in W447F mutant cells), which is likely BH4-deficient but still capable of reducing its heme and generating superoxide. This path would have significantly greater activity than flavin auto-oxidation in the monomer and, therefore, would overwhelm the small amount of superoxide produced by the monomer, despite the monomer being the major form of the eNOS enzyme present under this condition in the cell. This explains both the increased superoxide and the ability of L-NAME to inhibit the signal. These data are supported by our findings in C99A mutants. We now provide evidence that monomeric eNOS, produced by the mutation of C99A, is capable of producing superoxide in a BH4-independent manner. The lack of an effect of L-NAME is also striking. Production of superoxide from C99A eNOS appears to be different from that of WT or the W447 mutants.

The studies presented here are of great significance to advance our understanding of BH4 as a regulator of eNOS coupling, affecting signaling cascades dependent on NO and reactive oxygen species, and, importantly, as a therapeutic to restore endothelial function in vascular disease. Indeed, several studies have already explored the effect of BH4 administration, either intravascularly or orally, on endothelial functions. In clinical studies, pharmacological supplementation of BH4 improves endothelium-dependent relaxations and augments NO-mediated effects on forearm blood flow in smokers and those with diabetes and elevated cholesterol (30, 31, 39–41). These studies have been limited to acute or short-term administration, used very high doses, and only determined the effects on endothelial-dependent relaxation rather than other variables related to vascular disease progression or risk. Indeed, numerous studies have found that pharmacologic supplementation of BH4 augments NO-mediated effects in either cell culture or *in vitro* vessel rings, animal models, or patients with vascular disease risk factors (42, 43). Specifically, increasing BH4 biosynthesis in cultured endothelial cells, which are relatively BH4-deficient, restores eNOS activity and increases the proportion of eNOS protein present as the homodimeric form. Gene transfer of GTPCH in carotid arteries of DOCA-salt hypertensive rats restores BH4 levels and improves endothelial function (44), and when GTPCH is constitutively overexpressed specifically within endothelial cells in transgenic mice, tissue BH4 levels were increased and eNOS activity was restored (45). In GTPCH transgenic mice rendered diabetic with streptozotocin, the loss of vascular BH4 was prevented, leading to reduced evidence of eNOS uncoupling, and restored endothelial function. When GTPCH transgenic mice were crossed with ApoE KO mice, endothelial function was improved, and atherosclerotic plaque progression was reduced (29). Previous clinical studies from our group show that oral BH4 treatment in patients with coronary artery disease significantly elevates BH4 levels in blood, but this effect is significantly limited by systemic oxidation of exogenous BH4 to BH2, which lacks eNOS cofactor activity. Accordingly, the ratio of reduced to oxidized biopterins in blood and vascular tissue is unchanged by exogenous BH4 treatment, resulting in no net effect on eNOS coupling, endothelial function, or vascular superoxide production (46). Targeting BH4 is a rational therapeutic strategy in cardiovascular disease, but future studies should be directed toward interventions that can favorably alter the endogenous BH4/BH2 ratio and augment BH4 binding to eNOS in human vascular endothelium via a selective increase in absolute BH4 levels, prevention of BH4 oxidation, or increased BH4 recycling.

Because of the sensitivity of BH4 to oxidation, there is therapeutic potential for non-BH4, alternative pteridine analogues that possess more stable oxidative states. These compounds may further improve the impaired relaxation associated with endothelial dysfunction and substitute for BH4 within the vasculature. Two analogues of BH4 that can act as oxidatively stable alternatives to BH4, causing NO-mediated vasorelaxation, have been developed. Treatment with 6-hydroxymethyl pterin and 6-acetyl-7,7- dimethyl-7,8-dihydropterin improved endothelium-dependent vasorelaxation in isolated perfused lungs from both normoxic and hypoxic rats and increased eNOS expression in these rats (36). The development of future compounds to restore NOS function in vascular disease states must consider the structural effects of BH4 binding to eNOS W447 and the role of this residues in eNOS coupling for BH4-based strategies to be successful.

We propose that, rather than characterizing uncoupled eNOS as “dysfunctional,” uncoupling by mechanisms such as impaired BH4 binding, BH4 deficiency, or posttranslantionally by glutathione, are in fact a tightly regulated mechanism that renders eNOS as a redox “hub.” This would mean that Trp-447 is a critical residue that ultimately determines BH4 binding and BH4-dependent uncoupling, linking BH4 binding with of a plethora of targets and pathways that lie downstream of eNOS that have been demonstrated to be modulated by cellular redox state. Further studies using purified protein *in vitro* will give further insights into the role of Trp-447 on eNOS enzyme kinetics, electron flow, and triihydrobiopterin radical formation. Observations suggest that changes in redox status can exert a powerful influence on cellular homeostasis via eNOS, and further studies are required to elucidate the mechanisms for this redox-sensitive, downstream signaling.

## Footnotes

2 The abbreviations used are:

iNOS: inducible NOS
eNOS: endothelial NOS
BH4: tetrahydrobiopterin
BH2: 7,8-dihydrobiopterin
eGFP: enhanced GFP
DOX: doxycycline
GTPCH: GTP cyclohydrolase I
l-NAME: NG-nitro-l-arginine methyl ester.

## References

[1] Forstermann U. Sessa W. C. (2012) Nitric oxide synthases. Regulation and function. Eur. Heart J. 33, 829–837 21890489 10.1093/eurheartj/ehr304 PMC3345541

[2] Xie Q. Nathan C. (1994) The high-output nitric oxide pathway. Role and regulation. J. Leukocyte Biol. 56, 576–582 7525816 10.1002/jlb.56.5.576

[3] Bredt D. S. Ferris C. D. Snyder S. H. (1992) Nitric oxide synthase regulatory sites. Phosphorylation by cyclic AMP-dependent protein kinase, protein kinase C, and calcium/calmodulin protein kinase. Identification of flavin and calmodulin binding sites. J. Biol. Chem. 267, 10976–10981 1375933

[4] Stuehr D. J. Ikeda-Saito M. (1992) Spectral characterization of brain and macrophage nitric oxide synthases. Cytochrome P-450-like hemeproteins that contain a flavin semiquinone radical. J. Biol. Chem. 267, 20547–20550 1383204

[5] Masters B. S. McMillan K. Nishimura J. Martasek P. Roman L. J. Sheta E. Gross S. S. Salerno J. (1996) Understanding the structural aspects of neuronal nitric oxide synthase (NOS) using microdissection by molecular cloning techniques. Molecular dissection of neuronal NOS. Adv. Exp. Med. Biol. 387, 163–169 8794208 10.1007/978-1-4757-9480-9_22

[6] Masters B. S. McMillan K. Sheta E. A. Nishimura J. S. Roman L. J. Martasek P. (1996) Neuronal nitric oxide synthase, a modular enzyme formed by convergent evolution. Structure studies of a cysteine thiolate-liganded heme protein that hydroxylates l-arginine to produce NO as a cellular signal. FASEB J. 10, 552–558; Correction (1996) *FASEB J.* **10,** 1107 8621055 10.1096/fasebj.10.5.8621055

[7] Crabtree M. J. Smith C. L. Lam G. Goligorsky M. S. Gross S. S. (2008) Ratio of 5,6,7,8-tetrahydrobiopterin to 7,8-dihydrobiopterin in endothelial cells determines glucose-elicited changes in NO vs. superoxide production by eNOS. Am. J. Physiol. Heart Circ. Physiol. 294, H1530–H1540 18192221 10.1152/ajpheart.00823.2007 PMC2722919

[8] Vásquez-Vivar J. Kalyanaraman B. (2000) Generation of superoxide from nitric oxide synthase. FEBS Lett. 481, 305–306 11041680 10.1016/s0014-5793(00)02001-9

[9] Vásquez-Vivar J. Kalyanaraman B. Martásek P. Hogg N. Masters B. S. Karoui H. Tordo P. Pritchard K. A. Jr. (1998) Superoxide generation by endothelial nitric oxide synthase. The influence of cofactors. Proc. Natl. Acad. Sci. U.S.A. 95, 9220–9225 9689061 10.1073/pnas.95.16.9220 PMC21319

[10] Cosentino F. Hishikawa K. Katusic Z. S. Lüscher T. F. (1997) High glucose increases nitric oxide synthase expression and superoxide anion generation in human aortic endothelial cells. Circulation 96, 25–28 9236411 10.1161/01.cir.96.1.25

[11] Guzik T. J. Mussa S. Gastaldi D. Sadowski J. Ratnatunga C. Pillai R. Channon K. M. (2002) Mechanisms of increased vascular superoxide production in human diabetes mellitus. Role of NAD(P)H oxidase and endothelial nitric oxide synthase. Circulation 105, 1656–1662 11940543 10.1161/01.cir.0000012748.58444.08

[12] Heitzer T. Ylä-Herttuala S. Luoma J. Kurz S. Münzel T. Just H. Olschewski M. Drexler H. (1996) Cigarette smoking potentiates endothelial dysfunction of forearm resistance vessels in patients with hypercholesterolemia. Role of oxidized LDL. Circulation 93, 1346–1353 8641023 10.1161/01.cir.93.7.1346

[13] Landmesser U. Dikalov S. Price S. R. McCann L. Fukai T. Holland S. M. Mitch W. E. Harrison D. G. (2003) Oxidation of tetrahydrobiopterin leads to uncoupling of endothelial cell nitric oxide synthase in hypertension. J. Clin. Invest. 111, 1201–1209 12697739 10.1172/JCI14172 PMC152929

[14] Maier W. Cosentino F. Lütolf R. B. Fleisch M. Seiler C. Hess O. M. Meier B. Lüscher T. F. (2000) Tetrahydrobiopterin improves endothelial function in patients with coronary artery disease. J. Cardiovasc. Pharmacol. 35, 173–178 10672847 10.1097/00005344-200002000-00001

[15] Wang Z. Q. Wei C. C. Ghosh S. Meade A. L. Hemann C. Hille R. Stuehr D. J. (2001) A conserved tryptophan in nitric oxide synthase regulates heme-dioxy reduction by tetrahydrobiopterin. Biochemistry 40, 12819–12825 11669618 10.1021/bi011182s

[16] Aoyagi M. Arvai A. S. Ghosh S. Stuehr D. J. Tainer J. A. Getzoff E. D. (2001) Structures of tetrahydrobiopterin binding-site mutants of inducible nitric oxide synthase oxygenase dimer and implicated roles of Trp457. Biochemistry 40, 12826–12832 11669619 10.1021/bi011183k

[17] Klatt P. Schmidt K. Lehner D. Glatter O. Bächinger H. P. Mayer B. (1995) Structural analysis of porcine brain nitric oxide synthase reveals a role for tetrahydrobiopterin and l-arginine in the formation of an SDS-resistant dimer. EMBO J. 14, 3687–3695 7543842 10.1002/j.1460-2075.1995.tb00038.x PMC394443

[18] Crabtree M. J. Tatham A. L. Al-Wakeel Y. Warrick N. Hale A. B. Cai S. Channon K. M. Alp N. J. (2009) Quantitative regulation of intracellular endothelial nitric oxide synthase (eNOS) coupling by both tetrahydrobiopterin-eNOS stoichiometry and biopterin redox status. Insights from cells with Tet-regulated GTP cyclohydrolase I expression. J. Biol. Chem. 284, 1136–1144 19011239 10.1074/jbc.M805403200

[19] Tzeng E. Billiar T. R. Robbins P. D. Loftus M. Stuehr D. J. (1995) Expression of human inducible nitric oxide synthase in a tetrahydrobiopterin (H4B)-deficient cell line. H4B promotes assembly of enzyme subunits into an active enzyme. Proc. Natl. Acad. Sci. U.S.A. 92, 11771–11775 8524846 10.1073/pnas.92.25.11771 PMC40484

[20] McDonald D. M. Alp N. J. Channon K. M. (2004) Functional comparison of the endothelial nitric oxide synthase Glu298Asp polymorphic variants in human endothelial cells. Pharmacogenetics 14, 831–839 15608562 10.1097/00008571-200412000-00006

[21] Cai S. Alp N. J. McDonald D. Smith I. Kay J. Canevari L. Heales S. Channon K. M. (2002) GTP cyclohydrolase I gene transfer augments intracellular tetrahydrobiopterin in human endothelial cells. Effects on nitric oxide synthase activity, protein levels and dimerisation. Cardiovasc. Res. 55, 838–849 12176133 10.1016/s0008-6363(02)00460-1

[22] de Bono J. P. Warrick N. Bendall J. K. Channon K. M. Alp N. J. (2007) Radiochemical HPLC detection of arginine metabolism. Measurement of nitric oxide synthesis and arginase activity in vascular tissue. Nitric Oxide 16, 1–9 16647284 10.1016/j.niox.2006.03.008

[23] Zhao H. Joseph J. Fales H. M. Sokoloski E. A. Levine R. L. Vasquez-Vivar J. Kalyanaraman B. (2005) Detection and characterization of the product of hydroethidine and intracellular superoxide by HPLC and limitations of fluorescence. Proc. Natl. Acad. Sci. U.S.A. 102, 5727–5732 15824309 10.1073/pnas.0501719102 PMC556312

[24] Fink B. Laude K. McCann L. Doughan A. Harrison D. G. Dikalov S. (2004) Detection of intracellular superoxide formation in endothelial cells and intact tissues using dihydroethidium and an HPLC-based assay. Am. J. Physiol. Cell Physiol. 287, C895–C902 15306539 10.1152/ajpcell.00028.2004

[25] Heales S. Hyland K. (1989) Determination of quinonoid dihydrobiopterin by high-performance liquid chromatography and electrochemical detection. J. Chromatogr. 494, 77–85 2584347 10.1016/s0378-4347(00)82658-4

[26] Chen P. F. Tsai A. L. Wu K. K. (1995) Cysteine 99 of endothelial, nitric oxide synthase (NOS-III) is critical for tetrahydrobiopterin-dependent NOS-III stability and activity. Biochem. Biophys. Res. Comm. 215, 1119–1129 7488039 10.1006/bbrc.1995.2579

[27] Channon K. M. (2004) Tetrahydrobiopterin. Regulator of endothelial nitric oxide synthase in vascular disease. Trends Cardiovasc. Med. 14, 323–327 15596110 10.1016/j.tcm.2004.10.003

[28] Ozaki M. Kawashima S. Yamashita T. Hirase T. Namiki M. Inoue N. Hirata K. Yasui H. Sakurai H. Yoshida Y. Masada M. Yokoyama M. (2002) Overexpression of endothelial nitric oxide synthase accelerates atherosclerotic lesion formation in apoE-deficient mice. J. Clin. Invest. 110, 331–340 12163452 10.1172/JCI15215 PMC151086

[29] Alp N. J. McAteer M. A. Khoo J. Choudhury R. P. Channon K. M. (2004) Increased endothelial tetrahydrobiopterin synthesis by targeted transgenic GTP-cyclohydrolase I overexpression reduces endothelial dysfunction and atherosclerosis in ApoE-knockout mice. Arterioscler. Thromb. Vasc. Biol. 24, 445–450 14707037 10.1161/01.ATV.0000115637.48689.77

[30] Fukuda Y. Teragawa H. Matsuda K. Yamagata T. Matsuura H. Chayama K. (2002) Tetrahydrobiopterin restores endothelial function of coronary arteries in patients with hypercholesterolaemia. Heart 87, 264–269 11847169 10.1136/heart.87.3.264 PMC1767023

[31] Higashi Y. Sasaki S. Nakagawa K. Fukuda Y. Matsuura H. Oshima T. Chayama K. (2002) Tetrahydrobiopterin enhances forearm vascular response to acetylcholine in both normotensive and hypertensive individuals. Am. J. Hypertens. 15, 326–332 11991218 10.1016/s0895-7061(01)02317-2

[32] Heitzer T. Wenzel U. Hink U. Krollner D. Skatchkov M. Stahl R. A. MacHarzina R. Bräsen J. H. Meinertz T. Münzel T. (1999) Increased NAD(P)H oxidase-mediated superoxide production in renovascular hypertension. Evidence for an involvement of protein kinase C. Kidney Int. 55, 252–260 9893134 10.1046/j.1523-1755.1999.00229.x

[33] Stroes E. Kastelein J. Cosentino F. Erkelens W. Wever R. Koomans H. Lüscher T. Rabelink T. (1997) Tetrahydrobiopterin restores endothelial function in hypercholesterolemia. J. Clin. Invest. 99, 41–46 9011574 10.1172/JCI119131 PMC507765

[34] Sagami I. Sato Y. Daff S. Shimizu T. (2000) Aromatic residues and neighboring Arg-414 in the (6R)-5,6,7, 8-tetrahydro-l-biopterin binding site of full-length neuronal nitric-oxide synthase are crucial in catalysis and heme reduction with NADPH. J. Biol. Chem. 275, 26150–26157 10846172 10.1074/jbc.M000534200

[35] Stuehr D. Pou S. Rosen G. M. (2001) Oxygen reduction by nitric-oxide synthases. J. Biol. Chem. 276, 14533–14536 11279231 10.1074/jbc.R100011200

[36] Kunuthur S. P. Milliken P. H. Gibson C. L. Suckling C. J. Wadsworth R. M. (2011) Tetrahydrobiopterin analogues with NO-dependent pulmonary vasodilator properties. Eur. J. Pharmacol. 650, 371–377 20950600 10.1016/j.ejphar.2010.09.070

[37] Zou M. H. Shi C. Cohen R. A. (2002) Oxidation of the zinc-thiolate complex and uncoupling of endothelial nitric oxide synthase by peroxynitrite. J. Clin. Invest. 109, 817–826 11901190 10.1172/JCI14442 PMC150913

[38] Cai S. Khoo J. Mussa S. Alp N. J. Channon K. M. (2005) Endothelial nitric oxide synthase dysfunction in diabetic mice. Importance of tetrahydrobiopterin in eNOS dimerisation. Diabetologia 48, 1933–1940 16034613 10.1007/s00125-005-1857-5

[39] Heitzer T. Brockhoff C. Mayer B. Warnholtz A. Mollnau H. Henne S. Meinertz T. Münzel T. (2000) Tetrahydrobiopterin improves endothelium-dependent vasodilation in chronic smokers. Evidence for a dysfunctional nitric oxide synthase. Circ. Res. 86, E36–E41 10666424 10.1161/01.res.86.2.e36

[40] Heitzer T. Krohn K. Albers S. Meinertz T. (2000) Tetrahydrobiopterin improves endothelium-dependent vasodilation by increasing nitric oxide activity in patients with type II diabetes mellitus. Diabetologia 43, 1435–1438 11126415 10.1007/s001250051551

[41] Stroes E. S. Koomans H. A. de Bruin T. W. Rabelink T. J. (1995) Vascular function in the forearm of hypercholesterolaemic patients off and on lipid-lowering medication. Lancet 346, 467–471 7637480 10.1016/s0140-6736(95)91322-x

[42] Alp N. J. Channon K. M. (2004) Regulation of endothelial nitric oxide synthase by tetrahydrobiopterin in vascular disease. Arterioscler. Thromb. Vasc. Biol. 24, 413–420 14656731 10.1161/01.ATV.0000110785.96039.f6

[43] Katusic Z. S. (2001) Vascular endothelial dysfunction. Does tetrahydrobiopterin play a role? Am. J. Physiol. Heart Circ. Physiol. 281, H981–986 11514262 10.1152/ajpheart.2001.281.3.H981

[44] Zheng J.-S. Yang X.-Q. Lookingland K. J. Fink G. D. Hesslinger C. Kapatos G. Kovesdi I. Chen A. F. (2003) Gene transfer of human guanosine 5′-triphosphate cyclohydrolase I restores vascular tetrahydrobiopterin level and endothelial function in low renin hypertension. Circulation 108, 1238–1245 12925450 10.1161/01.CIR.0000089082.40285.C3

[45] Alp N. J. Mussa S. Khoo J. Cai S. Guzik T. Jefferson A. Goh N. Rockett K. A. Channon K. M. (2003) Tetrahydrobiopterin-dependent preservation of nitric oxide-mediated endothelial function in diabetes by targeted transgenic GTP-cyclohydrolase I overexpression. J. Clin. Invest. 112, 725–735 12952921 10.1172/JCI17786 PMC182196

[46] Cunnington C. Van Assche T. Shirodaria C. Kylintireas I. Lindsay A. C. Lee J. M. Antoniades C. Margaritis M. Lee R. Cerrato R. Crabtree M. J. Francis J. M. Sayeed R. Ratnatunga C. Pillai R. Choudhury R. P. Neubauer S. Channon K. M. (2012) Systemic and vascular oxidation limits the efficacy of oral tetrahydrobiopterin treatment in patients with coronary artery disease. Circulation 125, 1356–1366 22315282 10.1161/CIRCULATIONAHA.111.038919 PMC5238935

